# Contribution of chronic diseases to the mild and severe disability burden in Belgium

**DOI:** 10.1186/s13690-015-0083-y

**Published:** 2015-08-03

**Authors:** Renata T. C. Yokota, Johan Van der Heyden, Stefaan Demarest, Jean Tafforeau, Willma J. Nusselder, Patrick Deboosere, Herman Van Oyen

**Affiliations:** Department of Public Health and Surveillance, Scientific Institute of Public Health, Rue Juliette Wytsmanstraat 14, Brussels, 1050 Belgium; Department of Social Research, Interface Demography, Vrije Universiteit Brussel, Brussels, 1050 Belgium; Department of Public Health, Ghent University, Ghent, Belgium; Department of Public Health, Erasmus MC, Rotterdam, The Netherlands

**Keywords:** Severe disability, Mild disability, Activity of daily living, Mobility limitations, Chronic diseases, Belgium

## Abstract

**Background:**

Population aging accompanied by an increased longevity with disability has raised international concern, especially due to its costs to the health care systems. Chronic diseases are the main causes of physical disability and their simultaneous occurrence in the population can impact the disablement process, resulting in different severity levels. In this study, the contribution of chronic diseases to both mild and severe disability burden in Belgium was investigated.

**Methods:**

Data on 21 chronic diseases and disability from 35,799 individuals aged 15 years or older who participated in the 1997, 2001, 2004, or 2008 Belgian Health Interview Surveys were analysed. Mild and severe disability were defined based on questions related to six activities of daily living and/or mobility limitations. To attribute disability by severity level to selected chronic diseases, multiple additive hazard models were fitted to each disability outcome, separately for men and women.

**Results:**

A stable prevalence of mild (5 %) and severe (2–3 %) disability was observed for the Belgian population aged 15 years or older between 1997 and 2008. Arthritis was the most important contributor in women with mild and severe disability. In men, low back pain and chronic respiratory diseases contributed most to the mild and severe disability burden, respectively. The contribution also differed by age: for mild disability, depression and chronic respiratory diseases were important contributors among young individuals, while heart attack had a large contribution for older individuals. For severe disability, neurological diseases and stroke presented a large contribution in young and elderly individuals, respectively.

**Conclusions:**

Our results indicate that the assessment of the contribution of chronic diseases on disability is more informative if different levels of disability are taken into consideration. The identification of diseases which are related to different levels of disability – mild and severe – can assist policymakers in the definition and prioritisation of strategies to tackle disability, involving prevention, rehabilitation programs, support services, and training for disabled individuals.

**Electronic supplementary material:**

The online version of this article (doi:10.1186/s13690-015-0083-y) contains supplementary material, which is available to authorized users.

## Background

Information on disability is considered essential to understand and respond to the global phenomenon of population aging, especially due to its economic costs and impact in the quality of life of individuals. Chronic diseases are known to be the main cause of physical disability [1] and to reduce the autonomy of individuals in performing basic activities of daily living (ADL) [2]. The simultaneous occurrence of chronic diseases in individuals can have a different impact in the disablement process, resulting in different disability severity levels, which ranges from difficulty to inability to perform a task without assistance, i.e. dependence [3–7].

Several methods have been proposed to assess the disability burden in a population. For cross-sectional data, Nusselder and Looman recently proposed the attribution method to assess the disability burden [8, 9]. This method is based on multiple additive hazard models, which partition the disability prevalence into the additive contribution of diseases and conditions in the presence of comorbidity [9, 10].

Although the method has been widely used to assess the disability burden in several countries [8, 10–16], to our knowledge, the contribution of chronic diseases to different disability severity levels was not yet investigated. In our previous study, we determined the major contributors of the disability burden without distinguishing different disability severity levels in Belgium [16]. However, this distinction can be useful to assist policy makers in the definition of strategies to reduce the disability burden, as the assessment of dependence to perform ADLs can predict institutionalization, the need of home care services, and mortality [2, 6, 17].

The aim of this study was to assess the contribution of selected chronic diseases to the mild and severe disability burden in Belgium. Here, mild disability was considered a measure of difficulty in performing at least one ADL or mobility task, while severe disability was considered a measurement of dependence – inability to perform at least one ADL without assistance – or severe restriction in mobility.

## Methods

### Study population

The Belgian Health Interview Survey (BHIS) data from 1997, 2001, 2004, and 2008 were used in this analysis. The BHIS is a national household survey, repeated every 3–5 years, commissioned by all the ministers responsible for public health at the federal, regional, and community levels in Belgium. It is conducted by the Scientific Institute of Public Health in collaboration with Statistics Belgium [18]. A multistage sampling design with geographical stratification (regions and provinces) and clustering (municipalities and households) was applied to obtain a representative sample of the Belgian population, including elderly individuals living in nursing homes and homes for the elderly. Proxy interviews were mandatory for individuals aged < 15 years and allowed for individuals with severe mental or physical illness not able to reply themselves, if the selected person was not reachable for more than 1 month, and for individuals who refused to participate but allowed proxy answers. Each survey included approximately 10,000 individuals. The response rate was 59 % in 1997, 61 % in 2001, 61 % in 2004, and 55 % in 2008. Sampling weights were used to take into account the complex sample design. The data quality control was implemented in the fieldwork, data entry, and statistical analysis [19]. More details about the surveys methodology can be found elsewhere [18, 20].

**Fig. 1:**
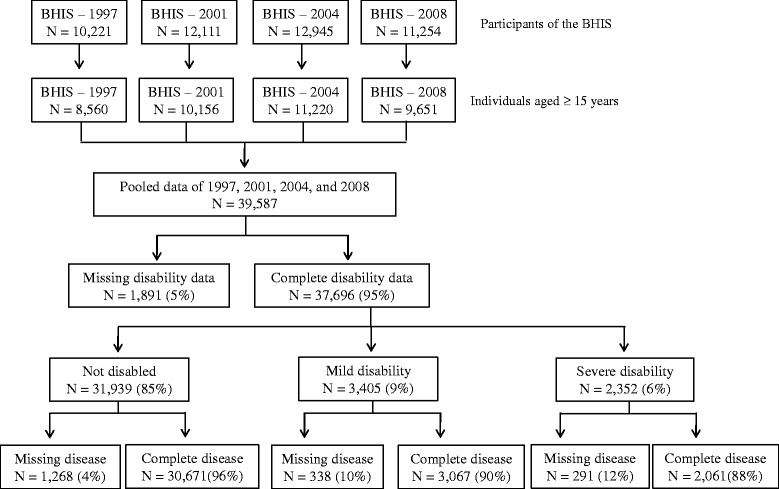
Description of the study sample. Health Interview Survey, Belgium, 1997, 2001, 2004, and 2008

This analysis was restricted to individuals aged 15 years or older, as the disability questions were restricted to this subpopulation. The selection of individuals included in this study is presented in Fig. 1. Subjects with missing information on disability or selected diseases (*n* = 3,788; 10 %) were excluded. In total, 35,799 individuals were included in the analysis (Fig. 1).

### Mild and severe disability

The definition of disability was based on self-reported questions about ADL and mobility limitations included in the face-to-face questionnaire in the four BHIS. For mobility limitations, the question “What is the furthest you can walk on your own without stopping and without severe discomfort?” was analysed, with possible answers: “a. Only a few steps”, “b. More than few steps, but less than 200 m”, or “c. 200 m or more”.

Six ADLs were included in the disability indicator: getting in and out of bed, getting in and out of chair, dressing and undressing, washing hands and face, feeding and cutting up food, and using the toilet. These questions are part of the instrument to measure disability proposed by the World Health Organization (WHO) [21]. Although urinary incontinence is also included in the set of questions recommended by the WHO [21], we did not include it in our disability definition, as it may exist without physical limitations and, therefore, it is no longer considered as ADL disability [6].

In the 1997, 2001, and 2004 BHIS, the questions “Can you … (ADL) on your own?”, with possible answers “a. Yes, without difficulty”, “b. Yes, with some difficulty”, “c. I only … (ADL) with someone to help me” were analysed. In 2008, the format of the ADL questions slightly changed, with one main question included before the list of ADLs: “Do you usually have difficulty doing any of these activities by yourself?” and one extra option of answer was included: “a. No difficulty”, “b. Yes, some difficulty”, “c. Yes, a lot of difficulty”, “d. I can’t achieve it by myself”.

A person was considered mildly disabled if he/she answered “b” to the mobility question or to at least one ADL question; and severely disabled if the answer was “a” to the mobility question or “c” to at least one ADL question in 1997, 2001, and 2004, or “c” or “d” to at least one ADL question in 2008.

As described above, the mild disability definition can be considered a measure of difficulty while the severe disability definition can be considered a measure of dependence.

### Chronic diseases

Data on self-reported chronic diseases in the year preceding the interview were collected through a face-to-face interview in the BHIS. In this analysis, 21 chronic diseases or disease groups that were available in the four BHIS were included: chronic respiratory diseases (asthma, chronic bronchitis, and chronic pulmonary diseases), diabetes, cancer, depression, chronic cystitis, heart attack, stroke, arthritis (rheumatoid arthritis and osteoarthritis), low back pain, osteoporosis, stomach ulcer, bowel diseases, liver diseases (hepatitis, cirrhosis, and other liver dysfunctions), gall-stones, cataract, glaucoma, migraine, thyroid problems, skin diseases, chronic kidney diseases, and neurological diseases (epilepsy and Parkinson’s disease).

Most of the diseases questions were not modified over the four BHIS waves. The main changes occurred for heart attack and low back pain. From 1997 to 2004, one question was included for “serious heart disease or heart attack”. However, in 2008, this question was split into two: “myocardial infarction” and “coronary heart diseases (angina pectoris)”. In this study, we grouped these two diseases, i.e. an individual was considered to have had a heart attack in the year preceding the interview if he/she answered “Yes” to the question of “serious heart disease or heart attack” in 1997–2004 or to at least to one of the questions – “myocardial infarction” or “coronary heart diseases (angina pectoris)” – in 2008.

In 1997, low back pain was described as “chronic spinal affection for longer than 3 months”, while in 2001 and 2004 “lumbago, sciatica, and disc prolapse” was added to the 1997 question. Nonetheless, in 2008, the question changed to “low back disorder or other chronic back defect”. The impact of the changes in the questions can be observed in the disease prevalence by gender and BHIS year, shown in the Additional file 1.

### Statistical analysis

Age and gender standardized disability prevalence per year using the direct standardization method [22] is presented to assess the disability prevalence trend for individuals aged 15 years or older in the study period.

The prevalence of disability by cause was estimated using the attribution method [11]. In this method, disability is attributed to disease and “background”, taking into account that individuals can have more than one disease (comorbidity) and that disability can be present in individuals without any disease [9]. For instance, even if an individual reports a disease in the survey, this is not necessarily the cause of the disability. This disability that is not associated with the diseases included in the analysis is labelled “background” [11].

The background may be a result of the disability that is not associated with any disease, underreporting and underdiagnosed diseases in the survey, disability causes that occurred before the year preceding the interview (for example, permanent consequences of accidents, falls), and disability causes not included in the survey or analysis [8–10].

The main assumptions of the method are: the distribution of disability by cause is entirely explained by diseases that are still present at the time of the survey and by the background; the cause-specific disability rates for each disease were proportionally equal in the time preceding the survey; individuals from the same age groups are exposed to the same background rate; the causes of disability (diseases and background) act as independent competing causes; and the start of the time at risk for disability is the same for all causes [9].

The attribution method is based on a multiple additive hazards model [11], defined as shown in (1).

1 $$\begin{array}{c}\hfill {Y}_i \sim Bernoulli\left({\pi}_i\right)\hfill \\ {}\hfill {\pi}_i=1-{e}^{-{\eta}_i}\hfill \\ {}\hfill {\eta}_i={\alpha}_a+{\displaystyle \sum_{d=1}^m}{\beta}_{cd}\times {X}_{di}\hfill \end{array}$$

Where *Y*_*i*_ is the binary response (disability) variable for each individual *i*; *π*_*i*_ is the estimated probability that individual *i* is disabled; *e* is the base of the natural logarithm; *η*_*i*_ is the total disability rate (linear predictor) for each individual *i*; $\alpha$_*a*_ is the background disability rate by age group *a*(1, …, *n*); *β*_*cd*_ is the disease-specific disability rate (disabling impact); and *X*_*di*_ is the indicator variable for each disease *d* and individual *i*.

In model (1), the disease-specific disability rate is defined as *β*_*cd*_ *= γ*_*c*_ × *δ*_*d*_, where *γ*_*c*_ is the age pattern, that varies across age group *c*(1, …, *k*) but is constant across diseases; and *δ*_*d*_ is the disease effect, which differs across diseases, but not by age group. In other words, both the background and the diseases are allowed to vary by age. Hence, model (1) is a reduced rank regression (RRR) model with one rank [23]. The attribution of disability to chronic diseases and background depends on the disease prevalence (*X*_*di*_) and on the disease-specific disability rate (*β*_*cd*_) [10].

The total disability rate (η_i_) is partitioned into background (α_a_) and disease-specific disability rates (β_cd_). The probability of individual *i* to be disabled due to disease $d$ is defined as ${\mathrm{D}}_{\mathrm{di}}=\frac{\upbeta_{\mathrm{cd}}\times {\mathrm{X}}_{\mathrm{di}}}{\upeta_{\mathrm{i}}}\times {\uppi}_{\mathrm{i}}$, i.e. the proportion of the disease-specific disability rate (β_cd_ × X_di_) in the total disability rate (η_i_) multiplied by the probability of being disabled (π_i_); and the probability of individual *i* to be disabled due to background is defined as ${\mathrm{B}}_{\mathrm{i}}=\frac{\upalpha_{\mathrm{a}}}{\upeta_{\mathrm{i}}}\times {\uppi}_{\mathrm{i}}$, i.e. the proportion of the background disability rate (α_a_) in the total disability rate (η_i_) multiplied by the probability of being disabled.

The total probability of being disabled for each individual $i$ is obtained by the sum of the cause-specific probabilities of being disabled: *B*_*i*_ and *D*_*di*_. The total number of disabled individuals by cause can be obtained by the sum of the cause-specific probabilities for each individual in the sample. The prevalence of disability by cause is then obtained by dividing the number of disabled individuals for each cause by the total number individuals in the sample.

For each disability outcome (mild and severe disability) separate models for men and women were fitted. The confidence intervals for the disease prevalence, parameter estimates of the models (background and disease-specific disability rates), and the prevalence of disability by cause were estimated by 1000 bootstrap replicas sampled with replacement of equal sample size as the original data [24].

The diseases that were not significant in the additive hazards models for each disability outcome were grouped as “other diseases” and included in the final models. The following non-significant diseases were included in the “other diseases” group: kidney diseases, liver diseases, gall-stones, glaucoma, cataract, thyroid problems, skin diseases, and migraine.

The multiple additive hazard models and the attribution of disability to chronic diseases were performed with the software developed by Nusselder and Looman [8, 9] in R, version 3.0.3 [25]. More details about the attribution method can be found in previous publications [8, 9, 11].

### Ethics

The surveys were carried out by Statistics Belgium within the legal framework provided by the statistical law in Belgium. Therefore, the project was exempted from submission to an ethical committee, but it had to be approved by the High Statistical Council. The use of the data by external researchers is possible upon authorisation from the Belgian Privacy Commission.

## Results

### Characteristics of the study population

Table 1 shows detailed information of the study population. A higher proportion of females, elderly (≥65 years), and low educated (no diploma or primary school) individuals were observed among mildly and severely disabled subjects compared to individuals without disability. Proxy interviews represented 9 % of the total number of interviews, reaching 29 % in the individuals with severe disability. The hospitalization rate in the 2004 and 2008 BHIS was 12.5 %, with an increasing trend with disability severity. Mobility limitation was the most frequent limitation, with increasing proportion according to disability severity level. For example, the proportion of mobility limitation was 77 % in severely disabled individuals and 65 % in mildly disabled individuals. The most common ADL limitations among individuals with mild disability were transfer in and out of bed (39.8 %) and transfer in and out of chair (35.5 %) while for severely disabled individuals, dressing and undressing (41.9 %) and transfer in and out of bed (35.5 %) were the most frequent ADL limitations.

**Table 1:** Characteristics of the study participants. Health Interview Survey, Belgium, 1997, 2001, 2004, and 2008

Characteristic	Total		Not disabled		Mild disability		Severe disability	
	*N*	%^a^	*N*	%^a^	*N*	%^a^	*N*	%^a^
Gender (female)								
Male	17019	47.5	15201	49.6	1177	38.4	641	31.1
Female	18780	52.5	15470	50.4	1890	61.6	1420	68.9
Age group (years)								
15–54	22486	62.8	21538	70.2	677	22.1	271	13.1
55–64	4490	12.5	3916	12.8	424	13.8	150	7.3
65–79	5634	15.7	4005	13.1	1097	35.8	532	25.8
≥80	3189	8.9	1212	4.0	869	28.3	1108	53.8
Survey year								
1997	7926	22.1	7085	23.1	586	19.1	255	12.4
2001	9175	25.6	8130	26.5	688	22.4	357	17.3
2004	9977	27.9	8293	27.0	971	31.7	713	34.6
2008	8721	24.4	7163	23.4	822	26.8	736	35.7
Education level								
Tertiary	11602	32.4	10711	34.9	615	20.1	276	13.4
Secondary	14145	39.5	12280	40.0	1194	38.9	671	32.6
Primary	5603	15.7	3737	12.2	1026	33.5	840	40.8
No diploma	512	1.4	304	1.0	105	3.4	103	5.0
No information	3937	11.0	3639	11.9	127	4.1	171	8.3
Proxy interview	3167	8.8	2339	7.6	226	7.4	602	29.2
Hospitalization rate^b^	2329	12.5	1521	9.8	373	20.8	435	30.2
Mobility limitation^c^	3797	10.6	-	-	2001	65.2	1592	77.2
ADL limitation^c^								
Transfer in/out of bed	2603	7.2	-	-	1220	39.8	731	35.5
Transfer in/out of chair	2378	6.7	-	-	1089	35.5	557	27.0
Dressing/undressing	2355	6.6	-	-	942	30.7	864	41.9
Washing hands and face	1171	3.3	-	-	255	8.3	508	24.6
Feeding	1151	3.2	-	-	265	8.6	492	23.9
Using the toilet	1210	3.4	-	-	251	8.2	511	24.8

^a^The percentages are not weighted: they do not represent the prevalence, but the proportion in the study population

^b^Inpatient hospitalization in the 12 months preceding the interview. Information available only for the 2004 and 2008 BHIS (*N* = 18,698)

^c^The proportions do not add to 100 %, as an individual can have more than one ADL and/or mobility limitation

### Age and gender standardized disability prevalence

**Fig. 2:**
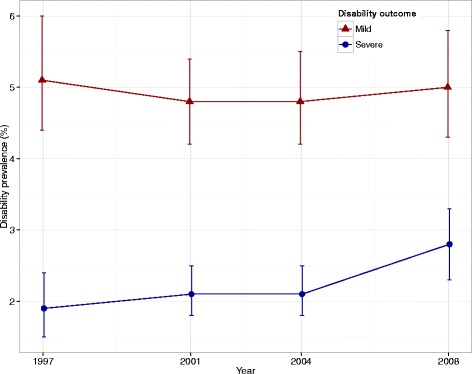
Age and gender standardized prevalence of mild and severe disability. Health Interview Survey, Belgium, 1997, 2001, 2004, and 2008

The age and gender standardized disability prevalence was higher for mild disability than for severe disability in the four BHIS (Fig. 2). However, no difference was observed for mild and severe disability over time across the four BHIS: the prevalence of mild disability varied from 5.1 % (95 % CI = 4.4 %; 6.0 %) in 1997 to 5.0 % (95 % CI = 4.3 %; 5.8 %) in 2008 and the prevalence of severe disability was 1.9 % (95 % CI = 1.5 %; 2.4 %) in 1997 and 2.8 % (95 % CI = 2.3 %; 3.3 %) in 2008 (Fig. 2).

### Disease prevalence

The attribution of disability to diseases and background is a function of two components: the disease prevalence and disease-specific disability rate. In general, the prevalence of chronic diseases was higher in individuals with mild or severe disability compared to non-disabled individuals (Tables 2 and 3). Arthritis and back pain were among the most prevalent diseases in men and women across all age groups and disability severity levels.

**Table 2:** Prevalence of chronic diseases in men according to disability severity. Health Interview Survey, Belgium, 1997, 2001, 2004, and 2008

Diseases	15–54 years			55–64 years			65–79 years			≥80 years		
	Not disabled	Mild	Severe	Not disabled	Mild	Severe	Not disabled	Mild	Severe	Not disabled	Mild	Severe
*n*	10806	284	131	1966	177	72	1905	440	188	524	276	250
Chronic respiratory diseases	4.9	13.4	10.2	7.9	22.0	30.3	12.3	30.1	28.6	11.8	25.3	36.3
Diabetes	1.1	3.5	4.9	5.8	10.3	27.7	10.1	14.6	8.4	8.0	11.6	14.7
Cancer	0.3	2.2	0.2	1.5	4.8	4.0	4.1	6.3	6.9	2.6	8.9	4.0
Depression	3.8	22.1	9.5	4.1	8.5	11.4	3.7	7.1	18.4	3.5	3.0	8.7
Neurological diseases	0.5	5.6	7.1	0.4	1.0	7.7	0.8	6.3	4.9	0.8	6.0	6.4
Stomach ulcer	2.2	10.0	5.2	3.9	16.1	6.1	4.6	8.9	7.2	4.2	9.1	4.6
Bowel diseases	1.7	8.4	6.6	2.8	5.6	6.4	3.6	4.4	6.0	3.9	5.3	9.7
Chronic kidney diseases	1.1	2.9	1.5	1.5	3.4	8.8	2.6	5.0	4.4	1.1	4.2	1.9
Liver diseases	0.5	4.1	0.0	0.9	0.5	0.5	0.7	4.4	0.5	0.3	1.2	1.4
Gall-stones	0.2	0.9	3.1	0.5	0.4	1.7	1.0	1.8	4.1	2.4	0.5	3.3
Glaucoma	0.8	2.8	2.8	1.9	3.9	5.2	3.3	7.2	4.5	4.3	5.2	8.5
Cataract	0.1	1.2	0.0	0.7	5.0	11.7	4.9	5.8	13.1	9.1	17.0	14.0
Migraine	5.7	19.7	9.8	4.5	8.7	12.6	2.9	4.4	4.3	3.0	5.5	6.6
Thyroid problems	0.7	2.3	2.7	2.2	5.5	1.0	2.6	4.3	7.5	2.1	3.0	2.0
Chronic skin diseases	2.8	5.1	5.5	2.5	7.2	6.2	4.0	2.3	5.1	3.1	4.9	9.0
Chronic cystitis	0.3	1.1	6.9	0.9	2.9	0.6	1.9	4.3	8.6	2.2	4.4	10.0
Cardiovascular diseases												
Heart attack	0.9	6.9	6.3	6.7	24.7	18.4	12.1	22.7	25.9	16.4	31.0	21.9
Stroke	0.2	1.6	3.0	0.5	2.9	9.9	1.6	1.3	8.4	3.0	7.1	9.9
Musculoskeletal diseases												
Low back pain	10.2	39.6	26.2	13.6	46.1	34.1	16.2	26.4	16.5	11.6	13.2	17.0
Osteoporosis	0.2	7.1	4.3	1.6	3.0	2.1	2.2	7.8	10.1	3.8	4.1	12.7
Arthritis	6.4	23.0	14.0	20.4	42.1	38.9	24.8	41.2	45.8	22.2	45.2	48.9

*n*: number of individuals

Arthritis: osteoarthritis and rheumatoid arthritis; chronic respiratory diseases: asthma, chronic bronchitis, chronic obstructive pulmonary disease, emphysema; neurological diseases: epilepsy and Parkinson’s disease

**Table 3:** Prevalence of chronic diseases in women according to disability severity. Health Interview Survey, Belgium, 1997, 2001, 2004, and 2008

Diseases	15–54 years			55–64 years			65–79 years			≥80 years		
	Not disabled	Mild	Severe	Not disabled	Mild	Severe	Not disabled	Mild	Severe	Not disabled	Mild	Severe
*n*	10732	393	140	1950	247	78	2100	657	344	688	593	858
Chronic respiratory diseases	6.0	20.7	15.4	6.9	16.0	15.2	7.9	21.3	24.4	7.9	10.5	15.1
Diabetes	1.2	5.0	3.1	4.9	12.2	14.0	8.0	12.2	18.3	5.6	8.9	11.1
Cancer	0.7	3.7	3.2	2.1	6.1	5.7	4.1	4.2	8.1	1.9	2.9	6.0
Depression	6.0	21.7	14.5	7.2	24.0	21.3	6.7	14.4	17.6	3.9	7.2	11.2
Neurological diseases	0.6	2.0	12.0	1.2	3.2	3.2	0.5	2.2	8.4	1.6	1.8	6.5
Stomach ulcer	2.2	9.5	2.7	4.3	9.8	18.0	4.3	9.7	10.1	3.4	5.7	10.7
Bowel diseases	2.4	14.1	6.9	3.7	9.3	18.7	4.7	11.0	15.2	1.8	7.4	7.7
Chronic kidney diseases	1.1	3.7	1.1	1.3	1.3	6.6	0.8	5.7	6.7	0.9	1.6	4.0
Liver diseases	0.4	1.7	1.0	0.8	1.7	2.1	1.5	2.1	2.6	0.2	1.5	2.0
Gall-stones	0.6	1.5	0.7	1.2	1.4	4.1	1.9	2.5	8.0	1.5	4.0	3.4
Glaucoma	0.6	1.0	0.7	3.1	9.0	6.1	4.9	7.5	10.0	5.4	8.8	8.3
Cataract	0.2	1.1	0.3	1.0	1.6	8.0	8.2	12.8	12.1	16.5	15.0	15.4
Migraine	15.5	29.8	24.6	10.9	26.9	25.7	7.8	13.5	18.8	5.9	5.7	9.9
Thyroid problems	4.6	11.5	7.8	8.8	9.5	10.4	9.5	12.3	14.6	4.7	10.7	12.0
Chronic skin diseases	3.4	5.7	0.4	2.9	8.2	2.9	3.2	4.9	3.8	3.2	1.5	7.5
Chronic cystitis	1.9	4.4	4.0	2.4	3.5	9.9	2.3	7.2	8.0	2.9	5.7	8.7
Cardiovascular diseases												
Heart attack	0.7	4.7	0.9	2.9	7.4	8.4	7.1	14.5	15.8	7.5	13.1	20.7
Stroke	0.1	2.7	4.0	0.9	2.7	5.1	1.0	4.0	7.6	2.1	2.6	9.5
Musculoskeletal diseases												
Low back pain	9.7	40.8	18.9	16.2	46.4	40.6	19.7	35.4	39.1	15.0	26.7	23.9
Osteoporosis	1.4	4.3	7.7	9.9	16.8	28.7	16.8	27.2	27.6	13.4	25.4	28.1
Arthritis	7.6	37.8	20.5	27.8	63.6	61.6	41.5	67.7	70.0	40.9	58.8	65.8

*n*: number of individuals

Arthritis: osteoarthritis and rheumatoid arthritis; chronic respiratory diseases: asthma, chronic bronchitis, chronic obstructive pulmonary disease, emphysema; neurological diseases: epilepsy and Parkinson’s disease

In disabled men, chronic respiratory diseases had a high prevalence in all age groups. For the youngest disabled men (15–54 years), depression and migraine were also among the most prevalent diseases. For instance, the prevalence of depression in mildly disabled young men was six times higher than in non-disabled men and two times higher than in severely disabled men. For men aged 55 or older, heart attack also had a high prevalence. While the prevalence of neurological diseases was very low in non-disabled men (<1 %) it reached 6.3 and 7.7 % in mildly and severely disabled men, respectively.

For women aged 15–64 years, migraine and depression were among the most frequent diseases. The prevalence of osteoporosis and chronic respiratory diseases was also high among women aged 55 or older. The prevalence of stroke was low (≤2 %) in non-disabled women, reaching 2.7 and 9.5 % in mildly and severely disabled women, respectively.

### Background disability rates

**Fig. 3:**
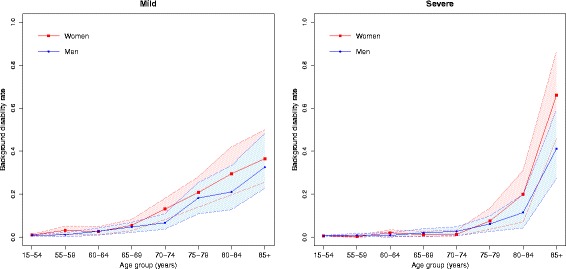
Background disability rate by gender and age groups. Health Interview Survey, Belgium, 1997, 2001, 2004, and 2008

**Fig. 4:**
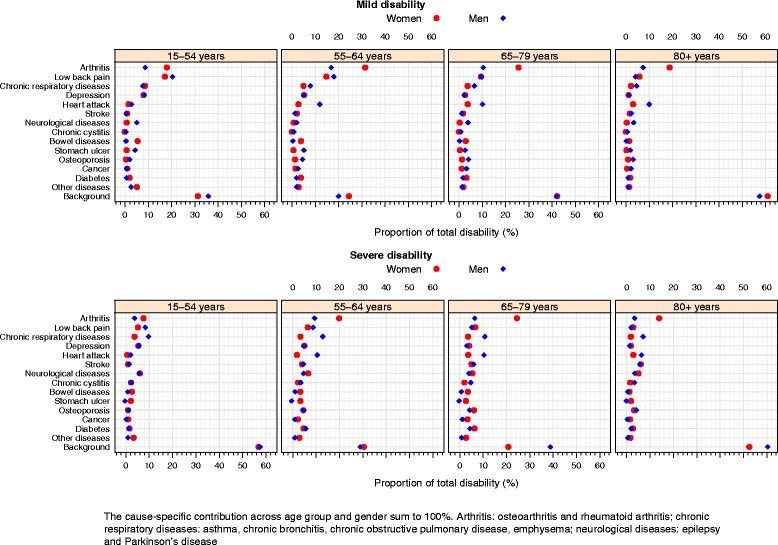
Relative contribution of diseases (proportion of total disability prevalence) to the prevalence of mild and severe disability. Health Interview Survey, Belgium, 1997, 2001, 2004, and 2008

The background disability rates, i.e., the disability rate related to the causes of disability that were not included in the analysis, are presented in Fig. 3. The background disability rates tend to increase over age groups, with a steep increase for severely disabled individuals aged 85 years or older. Although the background disability rates were low for young individuals (15–54 years), the relative contribution of background to the total disability prevalence was high, comparable to the background contribution of the oldest old (≥80 years), especially among the severely disabled individuals (Fig. 4).

### Disease-specific disability rates (disabling impacts)

The rank of the disabling impacts of the diseases (disease-specific disability rates) is presented in Tables 4 and 5 for men and women, respectively. Overall, the disease disability rates increased over age and were higher in women compared to men.

**Table 4:** Rank of disease-specific disability rates (disabling impacts) according to disability severity in men. Health Interview Survey, Belgium, 1997, 2001, 2004, and 2008

Rank	*Mild disability*											
	15–54 years			55–64 years			65–79 years			≥80 years		
	Diseases	Rate	95 % CI	Diseases	Rate	95 % CI	Diseases	Rate	95 % CI	Diseases	Rate	95 % CI
1	Neurological diseases	0.24	0.11;0.39	Neurological diseases	0.49	0.22;0.88	Neurological diseases	0.57	0.23;0.95	Neurological diseases	0.84	0.28;1.34
2	Osteoporosis	0.14	0.06;0.25	Osteoporosis	0.28	0.14;0.54	Osteoporosis	0.33	0.15;0.58	Osteoporosis	0.48	0.18;0.77
3	Stroke	0.08	0.00;0.19	Stroke	0.16	0.00;0.42	Stroke	0.19	0.00;0.42	Stroke	0.28	0.00;0.61
4	Cancer	0.07	0.01;0.15	Cancer	0.15	0.02;0.31	Cancer	0.18	0.02;0.32	Cancer	0.26	0.03;0.45
5	Heart attack	0.07	0.04;0.12	Heart attack	0.15	0.08;0.25	Heart attack	0.17	0.09;0.25	Heart attack	0.25	0.11;0.37
6	Depression	0.05	0.03;0.09	Depression	0.11	0.06;0.20	Depression	0.13	0.06;0.21	Depression	0.19	0.07;0.31
7	Low back pain	0.05	0.04;0.07	Low back pain	0.11	0.07;0.17	Low back pain	0.12	0.07;0.18	Low back pain	0.18	0.08;0.26
8	Stomach ulcer	0.05	0.02;0.08	Stomach ulcer	0.10	0.04;0.20	Stomach ulcer	0.12	0.04;0.22	Stomach ulcer	0.17	0.05;0.31
9	Chronic respiratory diseases	0.04	0.02;0.06	Chronic respiratory diseases	0.08	0.04;0.15	Chronic respiratory diseases	0.10	0.04;0.16	Chronic respiratory diseases	0.14	0.05;0.23
10	Arthritis	0.03	0.02;0.05	Arthritis	0.07	0.05;0.11	Arthritis	0.08	0.04;0.13	Arthritis	0.12	0.05;0.20
11	Diabetes	0.01	0.00;0.04	Diabetes	0.02	0.00;0.07	Diabetes	0.03	0.00;0.09	Chronic cystitis	0.10	−0.02;0.27
12	Chronic cystitis	0.03	−0.01;0.09	Other diseases^a^	0.01	0.00;0.04	Other diseases^a^	0.02	0.00;0.04	Diabetes	0.04	−0.01;0.12
13	Bowel diseases	0.01	−0.01;0.04	Chronic cystitis	0.06	−0.01;0.19	Chronic cystitis	0.07	−0.01;0.20	Bowel diseases	0.02	−0.03;0.13
14	Other diseases^a^	0.01	0.00;0.01	Bowel diseases	0.01	−0.02;0.09	Bowel diseases	0.01	−0.02;0.10	Other diseases^a^	0.02	−0.01;0.05
Rank	*Severe Disability*											
	55–64 years			55–64 years			65–79 years			≥80 years		
	Diseases	Rate	95 % CI	Diseases	Rate	95 % CI	Diseases	Rate	95 % CI	Diseases	Rate	95 % CI
1	Neurological diseases	0.12	0.05;0.21	Neurological diseases	0.30	0.10;0.52	Neurological diseases	0.34	0.15;0.71	Neurological diseases	0.72	0.27;1.31
2	Stroke	0.09	0.03;0.20	Stroke	0.23	0.08;0.40	Stroke	0.26	0.13;0.49	Stroke	0.54	0.24;0.98
3	Chronic cystitis	0.07	0.00;0.20	Chronic cystitis	0.18	0.01;0.35	Chronic cystitis	0.20	0.01;0.42	Chronic cystitis	0.42	0.02;0.85
4	Osteoporosis	0.05	0.00;0.13	Osteoporosis	0.12	0.01;0.24	Osteoporosis	0.14	0.01;0.32	Osteoporosis	0.29	0.02;0.62
5	Chronic respiratory diseases	0.02	0.01;0.04	Chronic respiratory diseases	0.06	0.02;0.10	Chronic respiratory diseases	0.07	0.03;0.13	Chronic respiratory diseases	0.14	0.05;0.27
6	Depression	0.02	0.00;0.04	Heart attack	0.06	0.01;0.10	Heart attack	0.06	0.02;0.14	Heart attack	0.14	0.04;0.25
7	Heart attack	0.02	0.01;0.05	Depression	0.04	0.00;0.10	Depression	0.05	0.00;0.18	Depression	0.10	0.00;0.29
8	Diabetes	0.01	0.00;0.03	Diabetes	0.03	0.00;0.07	Diabetes	0.04	0.01;0.08	Diabetes	0.08	0.01;0.17
9	Cancer	0.01	0.00;0.03	Low back pain	0.02	0.00;0.04	Low back pain	0.03	0.01;0.05	Low back pain	0.06	0.02;0.11
10	Low back pain	0.01	0.00;0.02	Arthritis	0.02	0.00;0.03	Arthritis	0.02	0.00;0.05	Arthritis	0.04	0.00;0.10
11	Bowel diseases	0.01	0.00;0.02	Cancer	0.02	0.00;0.06	Cancer	0.02	−0.01;0.08	Cancer	0.05	−0.01;0.15
12	Arthritis	0.01	0.00;0.02	Bowel diseases	0.01	-0.01;0.05	Bowel diseases	0.02	−0.01;0.07	Bowel diseases	0.03	−0.02;0.13
13	Other diseases^a^	0.00	0.00;0.01	Other diseases^a^	0.00	-0.01;0.02	Other diseases^a^	0.00	−0.01;0.03	Other diseases^a^	0.01	−0.02;0.05
14	Stomach ulcer	0.00	−0.01;0.00	Stomach ulcer	−0.01	-0.01;0.01	Stomach ulcer	−0.01	−0.02;0.01	Stomach ulcer	−0.02	−0.03;0.02

Arthritis: osteoarthritis and rheumatoid arthritis; chronic respiratory diseases: asthma, chronic bronchitis, chronic obstructive pulmonary disease, emphysema; neurological diseases: epilepsy and Parkinson’s disease

^a^Other diseases: kidney diseases, liver diseases, gall-stones, glaucoma, cataract, thyroid problems, skin diseases, and migraine

**Table 5:** Rank of disease-specific disability rates (disabling impacts) according to disability severity in women. Health Interview Survey, Belgium, 1997, 2001, 2004, and 2008

Rank	*Mild disability*											
	15–54 years			55–64 years			65–79 years			≥80 years		
	Diseases	Rate	95 % CI	Diseases	Rate	95 % CI	Diseases	Rate	95 % CI	Diseases	Rate	95 % CI
1	Stroke	0.16	0.05;0.37	Stroke	0.28	0.09;0.59	Stroke	0.34	0.10;0.74	Stroke	0.50	0.14;1.02
2	Arthritis	0.08	0.05;0.11	Arthritis	0.13	0.09;0.18	Arthritis	0.16	0.12;0.22	Arthritis	0.24	0.14;0.31
3	Heart attack	0.07	0.02;0.12	Bowel diseases	0.12	0.04;0.21	Bowel diseases	0.15	0.06;0.29	Bowel diseases	0.22	0.07;0.39
4	Bowel diseases	0.07	0.03;0.13	Heart attack	0.11	0.04;0.20	Heart attack	0.14	0.05;0.24	Heart attack	0.20	0.06;0.35
5	Low back pain	0.06	0.04;0.08	Low back pain	0.10	0.06;0.15	Low back pain	0.12	0.08;0.18	Low back pain	0.18	0.09;0.26
6	Chronic respiratory diseases	0.05	0.03;0.07	Diabetes	0.09	0.03;0.16	Diabetes	0.11	0.03;0.20	Diabetes	0.16	0.04;0.29
7	Diabetes	0.05	0.01;0.10	Chronic respiratory diseases	0.08	0.04;0.13	Chronic respiratory diseases	0.10	0.05;0.17	Chronic respiratory diseases	0.15	0.07;0.24
8	Cancer	0.04	0.00;0.10	Depression	0.08	0.03;0.13	Cancer	0.09	0.01;0.20	Depression	0.14	0.05;0.23
9	Depression	0.04	0.02;0.07	Cancer	0.07	0.01;0.17	Depression	0.09	0.04;0.17	Cancer	0.13	0.01;0.28
10	Other diseases^a^	0.01	0.00;0.02	Other diseases^a^	0.01	0.00;0.03	Other diseases^a^	0.02	0.00;0.04	Other diseases^a^	0.02	0.00;0.05
11	Neurological diseases	0.04	−0.01;0.13	Neurological diseases	0.07	−0.02;0.23	Neurological diseases	0.08	−0.02;0.29	Neurological diseases	0.12	−0.03;0.38
12	Osteoporosis	0.01	−0.01;0.03	Osteoporosis	0.02	−0.02;0.05	Osteoporosis	0.02	−0.02;0.07	Osteoporosis	0.03	−0.03;0.10
13	Stomach ulcer	0.01	−0.01;0.05	Stomach ulcer	0.02	−0.02;0.08	Stomach ulcer	0.02	−0.03;0.11	Stomach ulcer	0.03	−0.04;0.14
14	Chronic cystitis	−0.01	−0.01;0.03	Chronic cystitis	−0.02	−0.02;0.05	Chronic cystitis	−0.02	−0.03;0.06	Chronic cystitis	−0.03	−0.04;0.08
Rank	*Severe Disability*											
	15–54 years			55–64 years			65–79 years			≥80 years		
	Diseases	Rate	95 % CI	Diseases	Rate	95 % CI	Diseases	Rate	95 % CI	Diseases	Rate	95 % CI
1	Neurological diseases	0.12	0.04;0.27	Neurological diseases	0.22	0.11;0.45	Neurological diseases	0.77	0.35;1.24	Neurological diseases	1.61	0.94;3.12
2	Stroke	0.09	0.03;0.19	Stroke	0.17	0.07;0.34	Stroke	0.59	0.25;0.89	Stroke	1.23	0.68;2.26
3	Diabetes	0.02	0.01;0.03	Diabetes	0.03	0.01;0.07	Diabetes	0.11	0.04;0.18	Diabetes	0.22	0.10;0.46
4	Cancer	0.02	0.00;0.05	Cancer	0.03	0.00;0.10	Cancer	0.10	0.00;0.26	Cancer	0.22	0.01;0.70
5	Chronic respiratory diseases	0.01	0.00;0.02	Chronic cystitis	0.03	0.00;0.08	Chronic cystitis	0.09	0.01;0.22	Chronic cystitis	0.19	0.03;0.57
6	Depression	0.01	0.00;0.02	Bowel diseases	0.03	0.00;0.07	Bowel diseases	0.09	0.00;0.20	Bowel diseases	0.19	0.01;0.50
7	Chronic cystitis	0.01	0.00;0.04	Arthritis	0.03	0.01;0.05	Arthritis	0.09	0.04;0.12	Stomach ulcer	0.18	0.00;0.50
8	Heart attack	0.01	0.00;0.03	Depression	0.02	0.00;0.05	Depression	0.08	0.01;0.16	Arthritis	0.18	0.10;0.35
9	Low back pain	0.01	0.00;0.02	Heart attack	0.02	0.00;0.05	Stomach ulcer	0.08	0.00;0.19	Depression	0.16	0.02;0.42
10	Osteoporosis	0.01	0.00;0.02	Osteoporosis	0.02	0.00;0.04	Heart attack	0.07	0.00;0.15	Heart attack	0.14	0.00;0.42
11	Stomach ulcer	0.01	0.00;0.03	Stomach ulcer	0.02	0.00;0.07	Chronic respiratory diseases	0.05	0.01;0.12	Osteoporosis	0.11	0.02;0.26
12	Bowel diseases	0.01	0.00;0.03	Chronic respiratory diseases	0.01	0.00;0.04	Low back pain	0.05	0.01;0.09	Chronic respiratory diseases	0.10	0.01;0.30
13	Arthritis	0.01	0.00;0.03	Low back pain	0.01	0.00;0.03	Osteoporosis	0.05	0.01;0.10	Low back pain	0.10	0.01;0.23
14	Other diseases^a^	0.00	0.00;0.01	Other diseases^a^	0.00	0.00;0.01	Other diseases^a^	0.01	−0.01;0.03	Other diseases^a^	0.02	−0.02;0.10

Arthritis: osteoarthritis and rheumatoid arthritis; chronic respiratory diseases: asthma, chronic bronchitis, chronic obstructive pulmonary disease, emphysema; neurological diseases: epilepsy and Parkinson’s disease

^a^Other diseases: kidney diseases, liver diseases, gall-stones, glaucoma, cataract, thyroid problems, skin diseases, and migraine

In men, neurological diseases had the highest impact on mild and severe disability in all age groups. For mildly disabled men, osteoporosis, stroke and cancer also had a great impact on disability, while for severely disabled men, stroke, chronic cystitis, and osteoporosis were among the most disabling diseases. Low disabling impacts were observed for diabetes, chronic cystitis, other diseases, and bowel diseases in mildly disabled men and for bowel diseases, stomach ulcer, and other diseases in severely disabled men (Table 4).

In women, the diseases with great impact on mild disability were stroke, arthritis, bowel diseases, and heart attack. In contrast, for women with severe disability, neurological diseases, stroke, diabetes, and cancer had the highest disabling impacts. Chronic cystitis and stomach ulcer were the least disabling diseases in mildly disabled and back pain and other diseases had a low impact on severe disability (Table 5).

### Contribution of chronic diseases and background to the disability burden

The contribution of diseases and background to the disability burden and the disability prevalence is presented in Tables 6 and 7 for men and women, respectively. Although disability is present in all age groups, the prevalence of mild and severe disability is low for the youngest individuals (15–54 years). The prevalence of mild disability was higher than severe disability for individuals aged 15–79 years. However, among the oldest old (≥80 years), different patterns were observed: no difference in the prevalence of mild and severe disability was observed among men, whilst in women the prevalence of severe disability reaches substantial levels (51 %), exceeding the prevalence of mild disability in this age group.

**Table 6:** Rank of absolute contribution of diseases and background to the prevalence of disability, according to disability severity in men. Health Interview Survey, Belgium, 1997, 2001, 2004, and 2008

Rank	*Mild disability*											
	15–54 years			55–64 years			65–79 years			≥80 years		
	Diseases	%	95 % CI	Diseases	%	95 % CI	Diseases	%	95 % CI	Diseases	%	95 % CI
1	Background	0.92	0.91;0.92	Background	1.81	1.77;1.86	Background	7.86	7.57;8.17	Background	19.68	18.74;20.52
2	Low back pain	0.52	0.36;0.70	Low back pain	1.66	0.99;2.45	Arthritis	1.97	1.07;2.98	Heart attack	3.48	1.74;5.76
3	Arthritis	0.22	0.13;0.33	Arthritis	1.55	0.91;2.27	Heart attack	1.91	1.10;2.80	Arthritis	2.57	1.21;4.38
4	Depression	0.21	0.11;0.35	Heart attack	1.10	0.55;1.74	Low back pain	1.82	1.16;2.57	Chronic respiratory diseases	1.60	0.68;2.82
5	Chronic respiratory diseases	0.20	0.11;0.30	Chronic respiratory diseases	0.73	0.33;1.24	Chronic respiratory diseases	1.26	0.58;2.04	Low back pain	1.43	0.78;2.27
6	Neurological diseases	0.13	0.06;0.22	Depression	0.47	0.22;0.79	Osteoporosis	0.77	0.32;1.38	Neurological diseases	1.15	0.30;2.54
7	Stomach ulcer	0.11	0.04;0.19	Stomach ulcer	0.47	0.17;0.84	Neurological diseases	0.73	0.22;1.50	Osteoporosis	1.06	0.42;1.93
8	Heart attack	0.07	0.03;0.13	Osteoporosis	0.41	0.19;0.67	Cancer	0.60	0.10;1.18	Cancer	0.74	0.10;1.58
9	Osteoporosis	0.05	0.02;0.10	Cancer	0.24	0.04;0.49	Stomach ulcer	0.50	0.19;0.87	Stomach ulcer	0.66	0.20;1.29
10	Diabetes	0.02	0.00;0.04	Neurological diseases	0.18	0.07;0.32	Depression	0.44	0.22;0.72	Depression	0.40	0.14;0.78
11	Cancer	0.02	0.00;0.05	Stroke	0.12	0.00;0.33	Stroke	0.23	0.00;0.47	Stroke	0.82	−0.01;2.17
12	Chronic cystitis	0.01	0.00;0.03	Other diseases^a^	0.18	−0.05;0.47	Diabetes	0.32	−0.05;0.78	Other diseases^a^	0.33	−0.09;0.89
13	Stroke	0.01	0.00;0.03	Diabetes	0.17	−0.03;0.42	Other diseases^a^	0.25	−0.07;0.66	Diabetes	0.32	−0.05;0.88
14	Bowel diseases	0.01	−0.01;0.07	Chronic cystitis	0.07	−0.01;0.17	Chronic cystitis	0.15	−0.02;0.40	Chronic cystitis	0.21	−0.04;0.61
15	Other diseases^a^	0.07	−0.02;0.16	Bowel diseases	0.03	−0.05;0.24	Bowel diseases	0.04	−0.07;0.30	Bowel diseases	0.06	−0.10;0.41
-	Total disability prevalence	2.57	2.24;2.94	Total disability prevalence	9.16	7.37;11.11	Total disability prevalence	18.84	16.18;21.47	Total disability prevalence	34.51	29.91;39.84
Rank	*Severe Disability*											
	15–54 years			55–64 years			65–79 years			≥80 years		
	Diseases			Diseases	%	95 % CI	Diseases	%	95 % CI	Diseases	%	95 % CI
1	Background	0.64	0.64;0.64	Background	0.92	0.91;0.93	Background	3.18	3.09;3.27	Background	17.37	16.28;18.47
2	Chronic respiratory diseases	0.11	0.05;0.18	Chronic respiratory diseases	0.43	0.15;0.81	Chronic respiratory diseases	0.90	0.32;1.59	Chronic respiratory diseases	2.06	0.70;4.12
3	Low back pain	0.09	0.03;0.18	Heart attack	0.34	0.09;0.66	Heart attack	0.88	0.25;1.78	Heart attack	1.85	0.57;3.46
4	Depression	0.06	0.00;0.14	Arthritis	0.31	0.02;0.70	Arthritis	0.55	0.03;1.30	Stroke	1.63	0.65;3.01
5	Neurological diseases	0.06	0.03;0.11	Low back pain	0.29	0.07;0.60	Stroke	0.49	0.23;0.83	Osteoporosis	1.23	0.10;2.74
6	Arthritis	0.04	0.00;0.10	Diabetes	0.19	0.02;0.45	Low back pain	0.43	0.14;0.78	Arthritis	1.00	0.06;2.35
7	Chronic cystitis	0.03	0.00;0.08	Depression	0.16	0.00;0.42	Chronic cystitis	0.38	0.03;0.82	Neurological diseases	0.99	0.39;1.91
8	Heart attack	0.02	0.00;0.05	Stroke	0.15	0.05;0.31	Diabetes	0.36	0.05;0.70	Chronic cystitis	0.98	0.07;2.28
9	Stroke	0.02	0.00;0.04	Osteoporosis	0.15	0.01;0.31	Osteoporosis	0.35	0.03;0.82	Diabetes	0.57	0.06;1.26
10	Diabetes	0.01	0.00;0.04	Neurological diseases	0.15	0.04;0.35	Neurological diseases	0.33	0.12;0.57	Low back pain	0.56	0.18;1.11
11	Osteoporosis	0.01	0.00;0.03	Chronic cystitis	0.11	0.01;0.26	Depression	0.27	0.00;0.87	Depression	0.38	−0.01;1.09
12	Cancer	0.00	0.00;0.01	Cancer	0.03	−0.01;0.09	Cancer	0.10	−0.03;0.31	Bowel diseases	0.14	−0.09;0.60
13	Bowel diseases	0.01	−0.01;0.04	Bowel diseases	0.03	−0.02;0.15	Other diseases^a^	0.07	−0.19;0.48	Other diseases^a^	0.14	−0.34;0.94
14	Other diseases^a^	0.01	−0.04;0.08	Other diseases^a^	0.03	−0.10;0.22	Bowel diseases	0.06	−0.04;0.22	Cancer	0.11	−0.03;0.36
15	Stomach ulcer	0.00	−0.01;0.01	Stomach ulcer	−0.02	−0.04;0.02	Stomach ulcer	−0.03	−0.09;0.03	Stomach ulcer	−0.04	−0.13;0.07
-	Total disability prevalence	1.12	0.94;1.33	Total disability prevalence	3.28	2.24;4.39	Total disability prevalence	8.32	6.43;10.34	Total disability prevalence	28.97	24.96;33.96

The disease contribution do not sum to the total disability prevalence due to rounding

Arthritis: osteoarthritis and rheumatoid arthritis; chronic respiratory diseases: asthma, chronic bronchitis, chronic obstructive pulmonary disease, emphysema; neurological diseases: epilepsy and Parkinson’s disease

^a^Other diseases: kidney diseases, liver diseases, gall-stones, glaucoma, cataract, thyroid problems, skin diseases, and migraine

The relative background contribution was high for all disability severity levels and age groups in men and women (Fig. 4). For severely disabled individuals, more than 50 % of the disability burden was attributed to background in the youngest (15–54 years) and oldest (≥80 years) individuals (Fig. 4).

### Men - mild disability

For mildly disabled men aged below 65 years, low back pain and arthritis were the main contributors to the disability burden. These two musculoskeletal diseases accounted for 29 and 35 % for the disability burden in mildly disabled men aged 15–54 years and 55–64 years, respectively (Table 6 and Fig. 4). Depression and heart attack were also important contributors for mildly disabled men aged 15–54 years and 55–64 years, respectively. In mildly disabled men at older ages (≥65 years) arthritis, heart attack, low back pain, and chronic respiratory diseases contributed most to the disability burden. Stroke (<3 %) and bowel diseases (<0.5 %) showed a low contribution to the mild disability prevalence in men (Table 6 and Fig. 4).

### Men - severe disability

In men with severe disability, chronic respiratory diseases were the main contributor to the disability burden in all age groups. Disability prevalence attributable to chronic respiratory diseases varied from 13 % in men aged 55–64 years to 7 % in men aged 85 and older. In the youngest severely disabled men, low back pain, neurological diseases, and depression were also important contributors, while for men aged 55–79 years, arthritis and heart attack were among the main contributors. For the oldest severely disabled men (≥80 years) heart attack and stroke were also important contributors to the disability burden (Table 6 and Fig. 4).

### Women - mild disability

In women with mild disability, arthritis and low back pain were the main contributors to the disability prevalence in all age groups, accounting for 25–46 % of the disability burden (Table 7 and Fig. 4). Depression and chronic respiratory diseases were also important contributors in women aged less than 65 years. Disability attributed to depression varied from 8 % in women aged 15–54 years and 5 % in women aged 55–64 years. For women aged 65 years or older, heart attack and chronic respiratory diseases were among the main contributors to the disability burden. Stomach ulcer, chronic cystitis, and neurological diseases were not important contributors (Table 7 and Fig. 4).

**Table 7:** Rank of absolute contribution of diseases and background to the prevalence of disability, according to disability severity in women. Health Interview Survey, Belgium, 1997, 2001, 2004, and 2008

Rank	*Mild disability*											
	15–54 years			55–64 years			65–79 years			≥80 years		
	Diseases	%	95 % CI	Diseases	%	95 % CI	Diseases	%	95 % CI	Diseases	%	95 % CI
1	Background	1.08	1.08;1.08	Arthritis	3.67	2.59;4.88	Background	10.55	10.23;10.85	Background	24.72	23.86;25.65
2	Arthritis	0.63	0.42;0.91	Background	2.83	2.79;2.86	Arthritis	6.44	4.69;8.36	Arthritis	7.68	4.82;10.54
3	Low back pain	0.59	0.40;0.81	Low back pain	1.73	1.01;2.63	Low back pain	2.38	1.47;3.35	Low back pain	2.37	1.26;3.64
4	Chronic respiratory diseases	0.30	0.16;0.45	Depression	0.61	0.25;1.05	Chronic respiratory diseases	0.94	0.45;1.51	Heart attack	1.26	0.40;2.39
5	Depression	0.27	0.12;0.43	Chronic respiratory diseases	0.57	0.29;0.89	Heart attack	0.94	0.36;1.56	Chronic respiratory diseases	0.89	0.43;1.57
6	Bowel diseases	0.19	0.07;0.34	Bowel diseases	0.45	0.17;0.77	Diabetes	0.80	0.24;1.40	Diabetes	0.74	0.21;1.47
7	Diabetes	0.07	0.02;0.13	Diabetes	0.44	0.13;0.82	Bowel diseases	0.73	0.24;1.43	Stroke	0.68	0.20;1.32
8	Heart attack	0.05	0.02;0.09	Heart attack	0.32	0.11;0.57	Depression	0.66	0.26;1.21	Bowel diseases	0.56	0.19;1.05
9	Cancer	0.03	0.00;0.07	Stroke	0.24	0.07;0.49	Stroke	0.43	0.12;0.92	Depression	0.47	0.17;0.90
10	Stroke	0.03	0.01;0.08	Cancer	0.17	0.02;0.40	Cancer	0.30	0.04;0.64	Cancer	0.20	0.02;0.49
11	Other diseases^a^	0.18	−0.01;0.40	Other diseases^a^	0.33	−0.03;0.78	Osteoporosis	0.33	−0.32;1.11	Osteoporosis	0.38	−0.34;1.35
12	Stomach ulcer	0.02	−0.03;0.11	Osteoporosis	0.16	−0.15;0.51	Other diseases^a^	0.47	−0.05;1.09	Other diseases^a^	0.56	−0.04;1.35
13	Neurological diseases	0.02	−0.01;0.07	Neurological diseases	0.08	−0.03;0.27	Stomach ulcer	0.09	−0.12;0.46	Neurological diseases	0.13	−0.04;0.41
14	Osteoporosis	0.01	−0.01;0.05	Stomach ulcer	0.07	−0.09;0.35	Neurological diseases	0.07	−0.02;0.22	Stomach ulcer	0.08	−0.12;0.43
15	Chronic cystitis	−0.01	−0.03;0.05	Chronic cystitis	−0.01	−0.06;0.10	Chronic cystitis	−0.02	−0.09;0.18	Chronic cystitis	−0.02	−0.14;0.26
-	Total disability prevalence	3.47	3.04;3.90	Total disability prevalence	11.66	9.68;13.72	Total disability prevalence	25.10	22.32;27.93	Total disability prevalence	40.71	36.32;44.77
Rank	*Severe Disability*											
	15–54 years			55–64 years			65–79 years			≥80 years		
	Diseases			Diseases	%	95 % CI	Diseases	%	95 % CI	Diseases	%	95 % CI
1	Background	0.72	0.72;0.72	Background	1.13	1.08;1.18	Arthritis	3.25	1.84;4.68	Background	26.38	24.43;28.38
2	Arthritis	0.10	0.04;0.19	Arthritis	0.76	0.35;1.28	Background	2.74	2.60;2.89	Arthritis	7.08	3.54;11.35
3	Neurological diseases	0.08	0.02;0.21	Low back pain	0.25	0.03;0.55	Low back pain	0.87	0.11;1.73	Stroke	3.04	1.64;5.04
4	Depression	0.07	0.01;0.13	Neurological diseases	0.25	0.09;0.47	Diabetes	0.84	0.36;1.40	Neurological diseases	2.54	1.34;4.18
5	Low back pain	0.07	0.01;0.15	Depression	0.19	0.02;0.40	Osteoporosis	0.80	0.12;1.56	Osteoporosis	1.61	0.24;3.29
6	Chronic respiratory diseases	0.05	0.01;0.11	Diabetes	0.18	0.07;0.33	Neurological diseases	0.71	0.35;1.14	Heart attack	1.45	0.00;3.38
7	Chronic cystitis	0.03	0.00;0.07	Osteoporosis	0.17	0.02;0.39	Stroke	0.66	0.32;1.05	Low back pain	1.41	0.18;2.80
8	Stomach ulcer	0.03	0.00;0.06	Stroke	0.15	0.05;0.31	Depression	0.54	0.05;1.06	Diabetes	1.37	0.50;2.39
9	Bowel diseases	0.03	0.00;0.08	Chronic respiratory diseases	0.13	0.01;0.29	Chronic respiratory diseases	0.48	0.05;1.03	Depression	0.93	0.08;2.00
10	Diabetes	0.02	0.01;0.04	Stomach ulcer	0.13	0.00;0.29	Heart attack	0.48	0.00;1.01	Stomach ulcer	0.92	0.00;2.37
11	Cancer	0.01	0.00;0.03	Bowel diseases	0.13	0.00;0.30	Bowel diseases	0.46	0.02;1.07	Chronic respiratory diseases	0.89	0.11;1.90
12	Heart attack	0.01	0.00;0.02	Chronic cystitis	0.09	0.01;0.20	Cancer	0.44	0.02;1.00	Chronic cystitis	0.77	0.08;1.64
13	Stroke	0.01	0.00;0.03	Cancer	0.08	0.00;0.21	Stomach ulcer	0.35	0.00;0.76	Cancer	0.72	0.02;2.16
14	Osteoporosis	0.01	0.00;0.03	Heart attack	0.07	0.00;0.17	Chronic cystitis	0.26	0.03;0.57	Bowel diseases	0.61	0.03;1.37
15	Other diseases^a^	0.04	−0.03;0.13	Other diseases^a^	0.11	−0.06;0.32	Other diseases^a^	0.36	−0.26;1.00	Other diseases^a^	0.81	−0.54;2.34
-	Total disability prevalence	1.29	1.03;1.60	Total disability prevalence	3.80	2.67;5.03	Total disability prevalence	13.26	11.29;15.41	Total disability prevalence	50.51	45.45;56.07

The disease contribution do not sum to the total disability prevalence due to rounding

Arthritis: osteoarthritis and rheumatoid arthritis; chronic respiratory diseases: asthma, chronic bronchitis, chronic obstructive pulmonary disease, emphysema; neurological diseases: epilepsy and Parkinson’s disease

^a^Other diseases: kidney diseases, liver diseases, gall-stones, glaucoma, cataract, thyroid problems, skin diseases, and migraine

### Women - severe disability

Arthritis was the main contributor to the severe disability burden in women, accounting for 8–25 % of the total disability prevalence. For women at young ages (<65 years), neurological diseases, depression, and low back pain were also important contributors. For severely disabled women aged 65–79 years, low back pain and diabetes were among the main contributors, representing 7 and 6 % of the disability burden in this age group. In the oldest old women (≥80 years), stroke and neurological diseases had an important contribution to the disability burden, accounting for 6 and 5 %, respectively. Cancer and chronic cystitis had a low contribution to the severe disability prevalence in women (Table 7 and Fig. 4).

### Disability prevalence

The mild disability prevalence was higher than severe disability prevalence for men and women in all age groups, except in the oldest old women (≥80 years), in which the prevalence of severe disability was 1.2 times higher than the prevalence of mild disability.

The total mild disability prevalence was higher for women compared to men in all age groups. For severe disability, almost no gender difference was observed in individuals aged <65 years, while the prevalence of severe disability in women was 1.6 and 1.7 times higher than men aged 65–79 years and 80 years or older, respectively (Tables 6 and 7).

## Discussion

A stable prevalence of mild (5 %) and severe (2–3 %) disability was observed for the Belgian population aged 15 years or older between 1997 and 2008. For women, arthritis was the main contributor to the mild and severe disability burden. For men with mild disability, low back pain, arthritis, and heart attack contributed most to the disability burden in men aged 15–64 years, 65–79 years, and ≥ 80 years, respectively. For severely disabled men, chronic respiratory diseases contributed most to the disability prevalence. The contribution also differed by age: for mild disability, depression and chronic respiratory diseases were important contributors among young individuals, while heart attack had a large contribution for older individuals. For severe disability, neurological diseases and stroke presented a large contribution in young and elderly individuals, respectively.

### Disability prevalence

Different disability trends are found across countries and time [26, 27]. Although most studies in developed countries showed a decline in the disability prevalence [26, 28, 29], a stable disability prevalence was found across the four BHIS, similar to the trends in the Netherlands for activity limitations from 1990 to 2008 [30]. Despite the differences in the methodology and disability definition, the estimated prevalence of severe disability in Belgium was slightly higher than the one found in France in 2008–2009, where this prevalence was 1.4 % [12]. The small increase observed for severe disability in Belgium from 1997, 2001, and 2004 to 2008 can be due to the change in the options of answer in the ADL questions in 2008. In the BHIS from 1997 to 2004, only three answers were possible, while in the 2008 BHIS a fourth option – “with a lot of difficulty” – was added and included in our definition of severe disability.

The higher prevalence of mild compared to severe disability in all age groups indicates that difficulty is more common than dependence, except for women aged 80 years or older, in which more than half of the women reported dependence. Dependence is more common among women and increases with age [13, 31]. This gender difference can be a result of greater longevity and comorbidity in women compared to men [13, 31]. Another possible explanation for the gender difference is a composition effect. Among the oldest old individuals (≥80 years) in our study, 15 % of the men and 23 % of the women were aged 90 years or older. Since at the oldest ages women seem to spend more time severely disabled then men before death [32], the higher proportion of women aged 90 years or older compared to men in this study can also result in higher severe disability prevalence in women compared to men among the oldest old individuals.

Although young individuals are usually perceived as healthy [33], the mild and severe disability prevalence in this age group indicates that attention should also be given to the young individuals, to reduce future development and progression of disability.

It is interesting to notice that the most common ADLs in both mild and severe disabled individuals were lower extremity functions, such as limitations in mobility, transfer in and out bed and chair, although dressing and undressing difficulties are related to both lower and upper extremity functions [6]. This finding is line with the high contribution of arthritis observed in men and women across all age groups, as these lower extremity limitations involves the knee, a common site of arthritis [34, 35].

### Contribution of chronic diseases to the disability burden

The contribution of chronic diseases to the disability burden differs by gender, age, and disability severity level. Although reporting any difficulty in performing an ADL is considered a severe limitation, as ADLs are defined as basic tasks of daily life required for survival [26], longitudinal studies have shown that information on difficulty and dependence in performing ADLs are complementary. Thus, if both difficulty and dependence are assessed, they can better represent the continuum of disability than if severity level is ignored [36, 37]. Also, it has been demonstrated that a large proportion of the elderly individuals who report ADL is not homogenous, reinforcing the importance of assessing both, difficulty and dependence, to better understand this heterogeneity [37].

The contribution of diseases to the disability burden depends on the disease prevalence and the disease-specific disability rates [1, 10, 11]. For most of the main contributors, the attribution of disability was a function of high to moderate disease prevalence and low to moderate disabling impact. For example, among severely disabled women in all age groups, arthritis was by far the main contributor to the disability burden. The high prevalence of arthritis, reaching 70 % for women aged 65–79 years, combined with its low to moderate disabling impact (0.01–0.18) in severely disabled women, resulted in the identification of arthritis as the main contributor to the disability burden in all age groups. However, the most disabling diseases are not necessarily the most prevalent diseases [1]. For instance, stroke ranked second among the contributors to the severe disability burden in the oldest old women. For this group, stroke had a low prevalence (<10 %) compared to other diseases, but a very high disabling impact (1.23), resulting in an important contribution.

Low back pain and arthritis are known to be important causes of disability, especially among the elderly [8–14]. In our study these two musculoskeletal diseases were important contributors of mild disability in men and women and also among severely disabled women. Similar results were found in previous studies [8, 10–13, 15], although no distinction between mild and severe disability was reported in most of them, except for the study conducted in France. In the French study [12], instead of musculoskeletal diseases, psychiatric and neurological diseases had the largest contribution. A possible explanation for these differences is the difference in the disability definition, as the French study defined severe disability based exclusively on dependence on ADL, while in our study, the option “a lot of difficulty” was also incorporated to severe disability definition; in the definition of the disease groups, since dementia, headache, and multiple sclerosis were included in the neurological diseases group in the study conducted in France; and in the methodology, as the French study assessed the disability burden based on the average attribution fraction [38] to estimate the impact of diseases on disability, without stratifying the results per gender, hampering comparability.

In addition to musculoskeletal diseases, chronic respiratory diseases had an important contribution to the mild disability burden among young individuals. Chronic respiratory diseases have a direct effect on the development of disability in middle-aged individuals, with a great impact in the performance of lower extremity functions [39].

An interesting result is the association of depression with both mild and severe disability in young individuals, indicating its relation with difficulty and dependence. Depression is one of most disabling diseases, being the main cause of years of life lost due to disability in young individuals worldwide [12, 33, 40].

In general, the results for mild disability were very similar to our previous findings, using the same data (BHIS 1997, 2001, 2004, 2008) [16], but without distinguishing between disability severity level. Possibly, our previous results were driven by mild disability, since the number of individuals with mild disability was 1.5 times higher than that of severe disability.

Another important finding of this study was the identification of chronic respiratory diseases as the main contributor to the severe disability burden among men in all age groups. Although chronic respiratory diseases have been previously related to difficulty in mobility and in the performance of ADLs [10, 39, 41, 42] our findings suggest that chronic respiratory diseases are also an important cause of dependence in men. The relation of chronic respiratory diseases with dependence in ADLs was previously reported, and an increase with age and disease severity was observed [43].

Despite the different methodology used to assess the disability burden, a higher disabling impact for stroke compared to other diseases and an association of stroke with severe disability was also found in other studies [12, 44–48]. In our study it was an important contributor to severe disability in the oldest old individuals (≥80 years), suggesting a possible relation of stroke with dependence in this group.

Neurological diseases also had a high disabling impact for individuals with severe disability and for mildly disabled men. Additionally, neurological diseases were important contributors to the severe disability burden in the youngest (15–54 years) men and women and oldest (≥80 years) women, suggesting a relation with dependence in these groups. In our study, only Parkinson’s disease and epilepsy were included in the neurological diseases group. Parkinson’s disease [49, 50] and epilepsy [51] have been related to severe disability [12] and loss of autonomy. Parkinson’s disease is more common at older ages and, in this study, epilepsy seems to be responsible for most of the severe disability burden attributed to neurological diseases in young individuals.

Cancer was not an important contributor to the mild and severe disability burden. This is in agreement with previous studies [10, 12, 48]. One possible explanation for the low contribution of cancer is the high case-fatality rate observed in cancer patients combined with long periods of cancer prevalence without a substantial impact on ADLs [48].

The high background contribution observed in the youngest (15–54 years) and oldest (≥80 years) severely disabled individuals might indicate that important causes of severe disability in these groups were not included in our analysis, although 21 disease were initially considered in the models. Possible causes related to dependence in these groups are injuries, especially from road traffic accidents [33, 52], among the youngest, and mental disorders such as dementia [48, 53] among the elderly.

Although not assessed in this study, our attribution results might be related with risk factors such as smoking, obesity, and harmful alcohol use, as the diseases that contributed most to the disability burden – musculoskeletal, respiratory, and cardiovascular diseases – are also associated with these modifiable risk factors [7, 54–56]. Additionally, the increasing trend in the prevalence of these risk factors observed worldwide, especially at young ages may contribute to the disability burden [57]. While smoking is related with high mortality [58], obesity was identified as the most important risk factor associated with years lived with disability compared to smoking and harmful alcohol use in a study conducted in the Netherlands [59].

### Limitations and strengths

Some limitations should be carefully considered when interpreting the study results. Since we used cross-sectional data, we rely on the assumption that diseases cause disability. However, this is not necessarily true, although it is plausible [1] according to the disablement model proposed by Verbrugge and Jette [60]. Consequently, diseases may be incorrectly attributed to disability in cases where disability onset preceded disease onset. Also, the use of self-reported data may have resulted in an underestimation of the contribution of diseases, as the validity of self-reported diseases differs according to the diseases being studied [61]. Furthermore, the background contribution to both mild and severe disability might be overestimated due to lack of information of important disability causes, such as injuries and mental disorders [10].

Selection bias may also have occurred due to the low response rates in the four BHIS and due to the exclusion of individuals without disability or disease information (Fig. 1). We reported previously that individuals with missing information on disability and diseases were more likely to be women, elderly, and with low education level [16]. Therefore, our results might underestimate the true disability prevalence.

Another limitation is our definition of disability, which was based on functional and mobility limitations. Other measures of disability, including instrumental activities of daily living (IADL), cognitive impairments, and sensory limitations, such as hearing and vision limitations, also play an important role in the disablement process [62]. However, this information was not considered in our definition because these questions were not systematically included in all the four BHIS.

In general, proxy respondents tend to overestimate the disability prevalence and severity when compared to self-responders [6, 63, 64]. In our study, 8.8 % (*n* = 3167) of the interviews were answered by proxies, representing 10.4 % (*n* = 917) of the interviews with elderly individuals (≥65 years) (Table 1). Thus, an overestimation of the contribution of diseases to the disability burden might have occurred. However, the use of a mix of self-reporting and proxies is recommended in large surveys, especially those including elderly individuals [1, 64]. Our results support the use of proxies to investigate severe disability, as 29 % of the severely disabled individuals had a proxy interview (Table 1).

Although the attribution method allows the attribution of disability to more than one disease by including interaction terms between diseases in the model [9], we did not include interactions between diseases in our analysis, as it would result in a computer intensive task due to the large sample size (*N* = 35,799) and large number of diseases studied (*N* = 21 diseases). In the mortality analysis, a longitudinal extension of the average attribution fraction method has been proposed to take into account the coexistence of diseases in individuals [65, 66]. Both under and overestimation of the contribution of the diseases to the disability may have occurred in our study as a result of ignoring the interaction between diseases, similar to what is observed in the mortality analysis [65, 66].

Furthermore, the changes in the questions of some chronic diseases over the four BHIS impacted the prevalence of these diseases, resulting in a reduction in the prevalence of heart attack and increase in the prevalence of low back pain in 2008 compared with the previous surveys (Additional file 1). In addition to the stable mild and severe disability prevalence over time in the four BHIS (Fig. 2) and across age groups (Additional file 2), the difference in the disease prevalence of some diseases over time supports pooling the data of the four BHIS.

The use of a large sample is one of the strengths of the study, as it allowed the assessment of the contribution of chronic diseases to the disability burden across different disability severity levels. The inclusion of young individuals in this analysis is an added value of the study, as disability in young individuals is usually not investigated. Also, the representativeness of the sample used in this analysis was improved by including elderly individuals living in institutions. In our study, 6.6 % (*n* = 583) of the elderly (≥65 years) lived in institutions. Furthermore, the inclusion of depression in the analysis highlights the importance of assessing it in surveys, as it was identified as an important contributor to the mild and severe disability burden among young individuals. Another added value of this study is the attribution of disability to chronic diseases for different disability severity levels – moderate and severe.

## Conclusions

Different from several developed countries, the disability prevalence in Belgium based on functional and mobility limitations was stable from 1997 to 2008. The high contribution of musculoskeletal diseases to the mild and severe disability prevalence shows that intervention strategies to tackle these diseases can be attractive to reduce the disability burden, as they will have an impact on both difficulty and dependence.

Furthermore, focus should be given to chronic respiratory diseases, stroke, and neurological diseases, as they were among the most disabling diseases and contributors to the severe disability burden, indicating a relation of these diseases with dependence. Attention should also be given to depression, an important contributor to mild and severe disability among young individuals.

Our results indicate that the attribution of disability to chronic diseases is more informative if different levels of disability are taken into consideration. Measures of difficulty in ADL and mobility are generally used to evaluate health care treatment effectiveness. In contrast, dependence measures are used to identify demands for particular types of long-term care needs [1, 67]. In our study the hospitalization rate increased with disability severity level (Table 1), confirming previous findings [36]. Besides hospitalization, home care visits, nursing homes admission, and death also increase with disability severity level [36]. Therefore, the identification of which diseases are related to the different levels of disability severity can assist policymakers in the definition of strategies to tackle disability, involving prevention, rehabilitation programs (especially for dependence), support services, and training for disabled individuals [7].

## Additional files

Additional file 1:
**Disease prevalence according to gender and survey year.** Belgian Health Interview Survey, 1997, 2001, 2004, and 2008. Additional file 2:
**Prevalence of mild and severe disability across age groups and survey years.** Health Interview Survey, Belgium, 1997, 2001, 2004, and 2008.

## Abbreviations

ADL: Activity of daily living
BHIS: Belgian Health Interview Survey
IADL: Instrumental activities of daily living
RRR: Reduced rank regression
WHO: World Health Organization
