# Supplementary material for: Contribution of chronic diseases to the mild and severe disability burden in Belgium
Source: Arch Public Health. 2015 Aug 3;73(1):37. doi: 10.1186/s13690-015-0083-y (PMC4523000; doi:10.1186/s13690-015-0083-y)
Supplement: Additional file 1: — Disease prevalence according to gender and survey year. Belgian Health Interview Survey, 1997, 2001, 2004, and 2008. [file 13690_2015_83_MOESM1_ESM.docx]

### Additional file 1: Table S1 – Disease prevalence according to gender and survey year. Belgian Health Interview Survey, 1997, 2001, 2004, and 2008.

| Diseases | 1997 | | 2001 | | 2004 | | 2008 | |
| --- | --- | --- | --- | --- | --- | --- | --- | --- |
|  | % | 95%CI | % | 95%CI | % | 95%CI | % | 95%CI |
| *Men* |  |  |  |  |  |  |  |  |
| Chronic respiratory diseases | 7.2 | 6.1; 8.6 | 8.9 | 7.9; 9.9 | 8.4 | 7.4; 9.5 | 5.8 | 4.9; 6.7 |
| Diabetes | 2.3 | 1.9; 2.9 | 3.2 | 2.6; 3.8 | 3.6 | 3.0; 4.2 | 4.0 | 3.3; 4.8 |
| Cancer | 0.7 | 0.4; 1.1 | 1.3 | 1.0; 1.7 | 0.7 | 0.5; 0.9 | 1.8 | 1.3; 2.4 |
| Depression | 4.5 | 3.6; 5.5 | 4.7 | 3.9; 5.5 | 4.6 | 3.8; 5.5 | 4.1 | 3.2; 5.1 |
| Chronic cystitis | 0.8 | 0.5; 1.2 | 1.0 | 0.7; 1.4 | 1.2 | 0.8; 1.6 | 0.6 | 0.4; 0.8 |
| Chronic kidney diseases | 1.8 | 1.2; 2.4 | 1.4 | 1.0; 1.8 | 1.6 | 1.1; 2.1 | 1.4 | 1.0; 2.0 |
| Cardiovascular diseases |  |  |  |  |  |  |  |  |
| Heart attack | 4.4 | 3.5; 5.3 | 4.8 | 4.1; 5.6 | 5.3 | 4.5; 6.1 | 2.9 | 2.2; 3.6 |
| Stroke | 0.6 | 0.3; 0.9 | 0.7 | 0.5; 1.1 | 0.7 | 0.4; 1.0 | 0.7 | 0.5; 1.1 |
| Musculoskeletal diseases |  |  |  |  |  |  |  |  |
| Low back pain | 10.9 | 9.5; 12.4 | 11.1 | 9.9; 12.4 | 11.0 | 9.8; 12.2 | 17.6 | 16.0; 19.3 |
| Osteoporosis | 0.8 | 0.4; 1.2 | 1.5 | 1.0; 2.0 | 1.0 | 0.6; 1.4 | 1.3 | 0.9; 1.7 |
| Arthritis | 12.2 | 10.7; 13.8 | 13.1 | 11.8; 14.4 | 12.3 | 11.1; 13.7 | 11.7 | 10.4; 13.1 |
| Stomach ulcer | 2.9 | 2.2; 3.6 | 3.4 | 2.8; 4.0 | 2.8 | 2.3; 3.4 | 3.6 | 2.8; 4.7 |
| Bowel diseases | 2.6 | 1.9; 3.3 | 2.5 | 2.0; 3.1 | 2.7 | 2.1; 3.4 | 1.8 | 1.3; 2.4 |
| Liver diseases | 0.8 | 0.4; 1.2 | 0.7 | 0.4; 1.0 | 0.8 | 0.5; 1.2 | 0.5 | 0.3; 0.8 |
| Gall-stones | 0.3 | 0.1; 0.5 | 0.5 | 0.3; 0.8 | 0.6 | 0.3; 1.0 | 0.4 | 0.2; 0.6 |
| Glaucoma | 1.1 | 0.7; 1.6 | 1.8 | 1.3; 2.5 | 2.1 | 1.5; 2.6 | 1.1 | 0.8; 1.5 |
| Cataract | 0.7 | 0.5; 1.0 | 1.2 | 0.9; 1.6 | 1.5 | 1.2; 1.9 | 1.7 | 1.3; 2.3 |
| Migraine | 6.9 | 5.9; 8.0 | 6.0 | 5.1; 6.8 | 5.0 | 4.2; 5.9 | 4.6 | 3.8; 5.5 |
| Thyroid problems | 0.8 | 0.4; 1.3 | 1.5 | 1.1; 1.9 | 1.3 | 1.0; 1.8 | 1.6 | 1.1; 2.0 |
| Skin diseases | 3.0 | 2.4; 3.8 | 3.0 | 2.5; 3.6 | 3.9 | 3.0; 4.8 | 2.5 | 1.7; 3.5 |
| Neurological diseases | 0.7 | 0.3; 1.3 | 1.2 | 0.9; 1.7 | 1.2 | 0.8; 1.7 | 0.7 | 0.4; 1.0 |
| *Women* |  |  |  |  |  |  |  |  |
| Chronic respiratory diseases | 7.1 | 6.0; 8.2 | 9.0 | 7.9; 10.1 | 8.5 | 7.5; 9.5 | 7.2 | 6.2; 8.2 |
| Diabetes | 2.9 | 2.2; 3.7 | 3.2 | 2.6; 3.8 | 4.0 | 3.3; 4.7 | 4.1 | 3.4; 4.8 |
| Cancer | 1.5 | 0.9; 2.3 | 1.9 | 1.3; 2.6 | 1.5 | 1.1; 2.0 | 2.0 | 1.4; 2.6 |
| Depression | 8.2 | 7.2; 9.4 | 7.3 | 6.4; 8.3 | 7.0 | 6.1; 7.9 | 7.4 | 6.4; 8.4 |
| Chronic cystitis | 2.7 | 2.1; 3.4 | 2.6 | 2.1; 3.2 | 2.9 | 2.4; 3.5 | 2.1 | 1.5; 2.6 |
| Chronic kidney diseases | 1.6 | 1.1; 2.1 | 1.5 | 1.0; 2.0 | 1.6 | 1.1; 2.2 | 1.1 | 0.8; 1.5 |
| Cardiovascular diseases |  |  |  |  |  |  |  |  |
| Heart attack | 3.4 | 2.5; 4.5 | 3.8 | 3.1; 4.7 | 3.6 | 3.0; 4.2 | 2.0 | 1.4; 2.6 |
| Stroke | 1.2 | 0.6; 1.8 | 0.6 | 0.4; 0.9 | 0.7 | 0.5; 0.9 | 1.2 | 0.9; 1.6 |
| Musculoskeletal diseases |  |  |  |  |  |  |  |  |
| Low back pain | 12.1 | 10.8; 13.5 | 12.0 | 10.7; 13.2 | 11.7 | 10.5; 12.9 | 22.5 | 20.8; 24.3 |
| Osteoporosis | 4.8 | 3.9; 5.8 | 7.3 | 6.2; 8.6 | 6.5 | 5.7; 7.2 | 7.6 | 6.7; 8.6 |
| Arthritis | 18.5 | 16.6; 20.3 | 21.4 | 19.6; 23.1 | 18.3 | 17.1; 19.7 | 22.6 | 21.0; 24.3 |
| Stomach ulcer | 3.0 | 2.3; 3.8 | 4.2 | 3.5; 5.0 | 3.4 | 2.7; 4.2 | 3.6 | 2.9; 4.3 |
| Bowel diseases | 4.3 | 3.5; 5.1 | 4.2 | 3.3; 5.3 | 3.3 | 2.8; 3.9 | 3.7 | 3.0; 4.5 |
| Liver diseases | 1.0 | 0.6; 1.4 | 1.0 | 0.6; 1.5 | 0.5 | 0.3; 0.7 | 0.5 | 0.3; 0.7 |
| Gall-stones | 1.2 | 0.8; 1.8 | 1.1 | 0.8; 1.5 | 1.3 | 1.0; 1.7 | 0.9 | 0.6; 1.2 |
| Glaucoma | 1.5 | 1.0; 2.2 | 2.6 | 2.1; 3.3 | 2.4 | 1.8; 2.9 | 2.0 | 1.5; 2.6 |
| Cataract | 1.8 | 1.3; 2.5 | 2.5 | 2.0; 3.0 | 2.7 | 2.2; 3.2 | 3.3 | 2.7; 4.0 |
| Migraine | 15.5 | 13.9; 17.1 | 15.2 | 14.0; 16.6 | 12.6 | 11.4; 13.8 | 14.4 | 13.0; 15.9 |
| Thyroid problems | 5.1 | 4.2; 6.3 | 6.3 | 5.3; 7.4 | 6.8 | 6.0; 7.6 | 7.3 | 6.2; 8.4 |
| Skin diseases | 3.6 | 2.8; 4.7 | 3.8 | 3.2; 4.5 | 3.7 | 3.0; 4.5 | 3.0 | 2.4; 3.7 |
| Neurological diseases | 1.1 | 0.6; 1.8 | 0.9 | 0.6; 1.2 | 1.0 | 0.7; 1.4 | 1.4 | 0.9; 2.0 |

Arthritis: osteoarthritis and rheumatoid arthritis; chronic respiratory diseases: asthma, chronic bronchitis, chronic obstructive pulmonary disease, emphysema; neurological diseases: epilepsy and Parkinson’s disease.
