# Supplementary figures and images for: Contribution of chronic diseases to the mild and severe disability burden in Belgium
Source: Arch Public Health. 2015 Aug 3;73(1):37. doi: 10.1186/s13690-015-0083-y (PMC4523000; doi:10.1186/s13690-015-0083-y)

## Mild

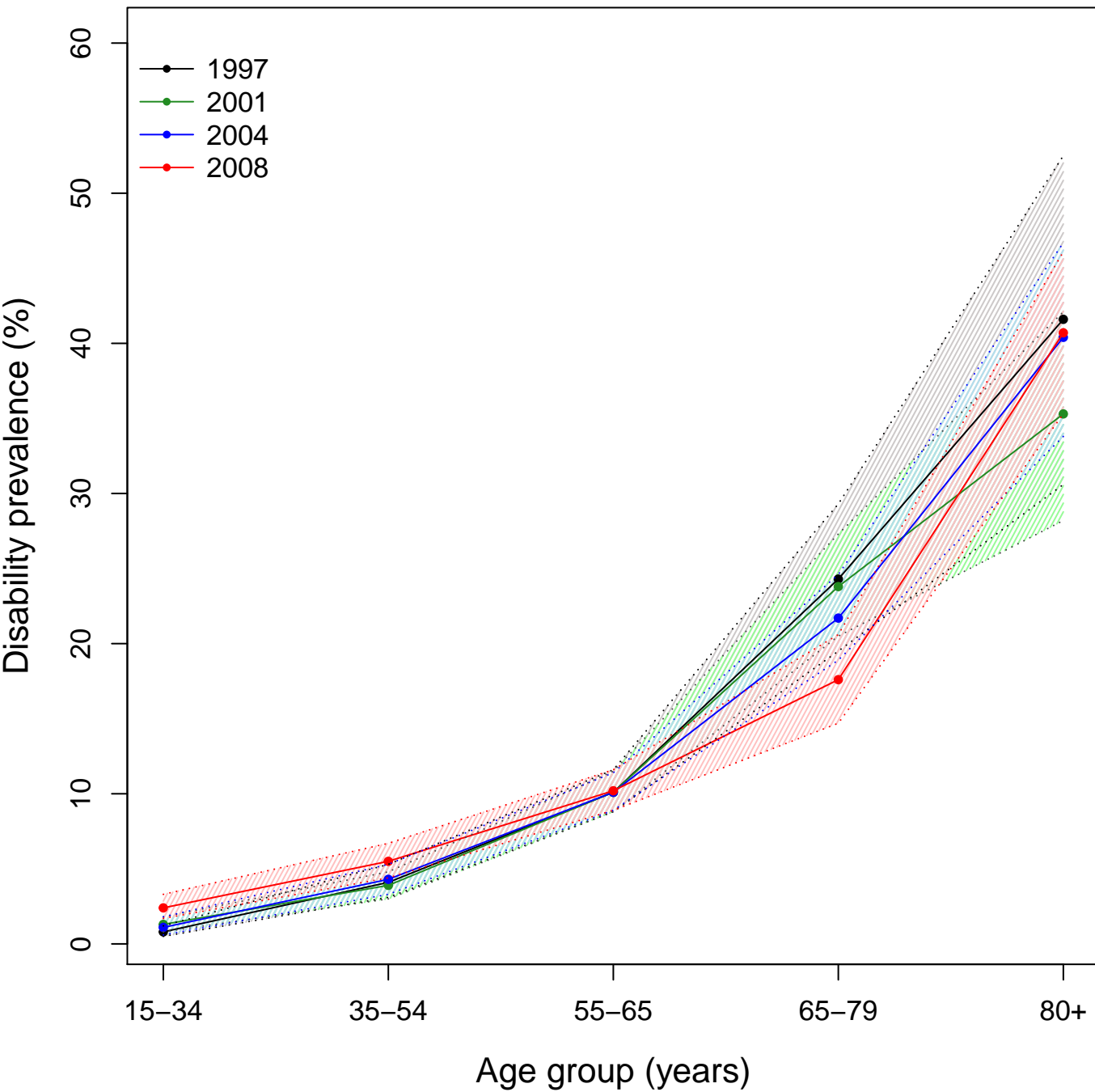

## Severe

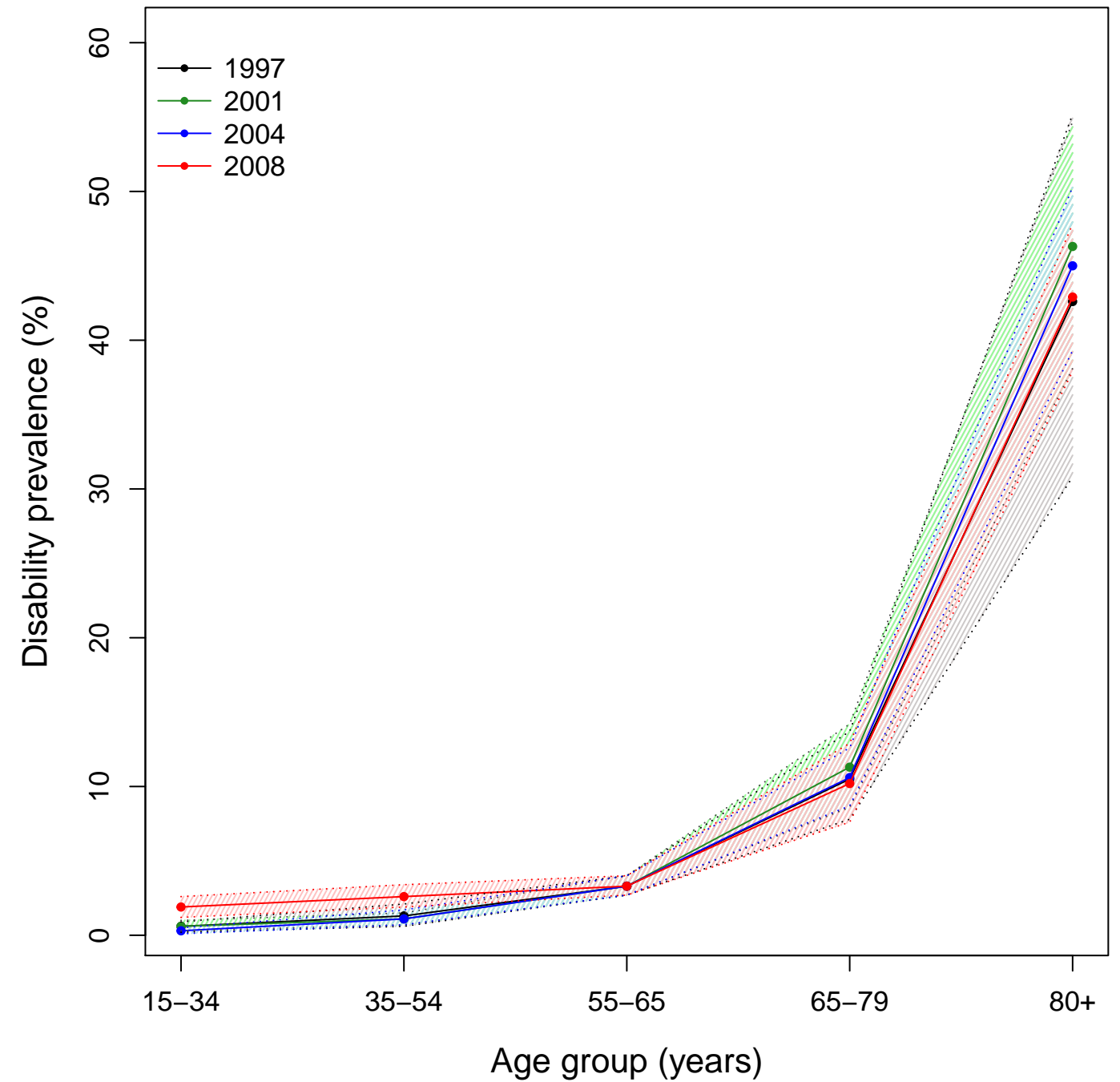

Supplement: Additional file 2: — Prevalence of mild and severe disability across age groups and survey years. Health Interview Survey, Belgium, 1997, 2001, 2004, and 2008. [file 13690_2015_83_MOESM2_ESM.pdf]
